# Lecture upon Homœopathy

**Published:** 1891-02

**Authors:** Walter S. Hatfield

**Affiliations:** Cincinnati, Ohio


### LECTURE UPON HOMŒOPATHY.

(Before the Students of Pulte Medical College.)

WALTER S. HATFIELD, M. D., CINCINNATI, OHIO.

GENTLEMEN:—This branch of study, the *Organon,* embodies the principles of Homœopathy. The rules by which, if strictly followed, we may the more certainly fulfill our life-work. The only method by which disease can be obliterated from the system the most gently, safely, and permanently.

In the introduction we see the absurdities and inconsistencies of old medicine, and Homœopathy can be credited with the burying out of sight of many of those antiquated methods and practices. People have been educated up to a higher plane. They expect to be cured of their ailments without so often being killed in the attempt.

Hahnemann gained the ill-will of the physicians of his day, because he dared to criticise the long-established methods and practices in medicine. But as truth and right constituted his motto he cared nothing for the slurs of those who differed from him, and we should be no less courageous, and besides, we have a fair portion of the people to aid us in the battle for the right.

By reading the introduction to the *Organon* we may get a fair idea of what the practice of medicine consisted at the time the *Organon* was written. The first edition was issued in 1810

and the fifth (last) edition in 1833. Thus you see, not many years have passed since to be sick meant sure death, or very nearly so in every case, if a doctor happened to be called in.

Previously to the establishment of Homœopathy everybody was bled. Lances, leeches, and cups were used to reduce the amount of blood within the system, and besides the patient must be vomited, purged, salivated, etc. But now conditions have changed. So much for Homœopathy, if nothing more. When we are sick unto death and unable to be cured, we are more liable to die on account of the disease than the treatment nowadays.

The old school still continues to appropriate our strength, as it were. Accidentally discovering something the homœopaths have been using many years perhaps, they take unto themselves the credit of a wonderful discovery.

Think of an allopath prescribing Hepar-sulph-calc. in trituration for otorrhœa! That was a wonderful discovery, which occurred but a few years ago, while the homœopaths have been using Hepar-sulph. a long time in such troubles, when indicated. But it was only a few years ago that an old school aurist found it useful in discharges from the ear, and so recommended it in the journals.

Many of their discoveries are of a similar character. But these discoveries and advanced ideas come from the *leaders* of the opposite school only, and it will take them a long time to influence the majority.

It is a hard matter to change a man's ideas and methods after they have once become established in his mind. Furthermore, if his father before him was a physician, he has had the influence of his father's staid qualities, and even though his father were not a physician his family was brought up in the good old way of the 3 P's., and of course there is nothing to do but follow the line of his boyhood education; and, besides, everybody expects just such treatment as that, and it is not difficult. I doubt not if we were to go back into the interior of some of the States we would find there old gray-headed doctors treating their patients just as they did many years ago, and if anybody would try to introduce a different method in his old-time families it would be found difficult, if not quite impossible. However, the more enlightened people are not so hide-bound.

But the prejudice of the old-school doctor is unbounded. It will not allow him to see the good results of Homœopathy, and the popular theme in the old school to-day is ridicule of Homœopathy. It is a dangerous rival, and how is it to be gotten rid of? is the question. They will not do as Dr. Hering did, "Read it up to write it down." And, besides, a portion of the people are too well-posted regarding the results of the homœopathic practice to allow it to be trampled under foot.

Dr. Hering was an honest man. He believed Homœopathy was wrong, and in an honest way he went about to prove it. He began by studying it thoroughly, so that he might prove it to be false, but in that investigation he became convinced of the truth of the homœopathic law. And ever afterward was, as you all know, one of its staunchest supporters and one of the brightest lights in our school. So much for honest investigation. Many of the strongest homœopaths of to-day were formerly allopathic practitioners, and, like Dr. Hering, were led out into the light after investigation, brought about by accident generally. And now that you have entered upon this field of life-work it should be your aim to learn aright.

Homœopathy is either all right or all wrong; to make a success of the practice of Homœopathy, one must understand the *Organon.* We cannot depend upon guess work and make a thoroughly proper homœopathic prescription.

In the introduction, Hahnemann mentions the fact that, for many centuries, many systems of medicine, or rather, many methods of treating the sick, have been brought forward, but not with any degree of success. About the time the new system would become started, some other method would be brought out, and so on, even until the present time.

But of all the different methods Hahnemann mentions more definitely allopathy in the introduction. And allopathy with all the rest fails to be in harmony with nature and experience in the cure of disease.

For many centuries old-school medicine has been in vogue, and for this reason allopaths assume their practice to be the nearest perfection because of the many centuries of experiment and experience.

But of what kind of experience can they boast? Every decade finds many changes made in their methods.

Their claim is that the causes of disease must be found and removed; which is impossible.

What is the cause of malaria, scarlatina, variola, etc.? The old school say it is bacteria! Everything is bacteria. A few years hence it will be something else.

Hahnemann's theory of a hundred years ago seems more plausible. But we will come to that later on.

The old-school members base their treatment upon the pathological condition. The symptoms, to them, are of no consequence, only so far as they may aid in the diagnosis. What is the name of the disease? is the all-important question with them.

After the patient is examined and a conclusion is arrived at, the treatment depends upon that conclusion. How difficult and uncertain the diagnosis is in many cases is apparent to any one who practices medicine. And to base all treatment upon an uncertain conclusion comes far from being rational. The discoveries made from post-mortem examinations are treated with great respect by allopaths, because, to them, the pathological changes mean much, while in reality, the only result is the effect following that mysterious cause—"the invisible disease power."

How often does the diagnosis prove incorrect when followed by a post-mortem, and consequently the treatment was wrong? And how often one hears the expression, "The patient died of a certain disease, and the doctors treated him for some other." But a homœopath cannot make that mistake, because he does not treat according to the name of the disease. Don't understand me to say that no diagnosis should be made, for I do not! But I do say that the symptoms, the totality of the symptoms, and not the diagnosis should be the guide in the treatment. Some physicians who are considered homœopaths will give certain remedies in pneumonia, typhoid fever, malaria, scarlatina, and rheumatism, etc., simply because the disease happens to be one or the other. But this comes far from being Homœopathy.

With some professed homœopaths as well as allopaths, the symptoms are serviceable only so far as they may aid in the diagnosis. And when the diagnosis is settled upon, the favorite prescription is given, no matter what the totality of the symptoms may be.

That is one great draw-back to Homœopathy, and this class of homœopaths generally give Morphia to relieve pain, etc., etc., because they have not the time to make a homœopathic prescription. Perhaps some of you may conclude from this that it is much more difficult and tedious to prescribe homœopathically. In some cases it may be, but not in all.

The first prescription may be more difficult, but afterward, if the first prescription be correct, the sailing will be smooth.

How often do we hear the remark: "I don't see any difference between the homœopaths and allopaths, they both give Morphia in pain, Quinine in malaria, etc."

When a man goes into a strange place and wishes to employ a homœopathic physician, if he knows anything about true Homœopathy, he is perplexed (for every professional homœopath is not homœopathic in truth, and more's the pity, for if they only were, Homœopathy would be more respected to-day), the one whom he chances to choose may prove to be of a liberal mind, and if the patient has a pain, very likely will get a dose of Morphia; and, after all, he has received just what he didn't want, for if he is acquainted with Homœopathy he is led to expect something better than that which he would get from the opposite school.

The only way to learn Homœopathy correctly is to study the *Organon.* And the only way to heal the sick gently, speedily, and permanently is to follow the teachings of the *Organon.*

We find in looking over the introduction that Hahnemann was not the first to observe that "Like cures like." One ancient writer observes the fact that the purging qualities of Rhubarb are the cause of its power to allay diarrhœa.

Another, that colic is cured by the infusion of Senna, because it produces colic in the healthy. Stahl, a Danish military physician, concluded thus : "The rule accepted in medicine, to cure by contraries, is entirely wrong." He is convinced, on the contrary, that diseases vanish and are cured by means of medicines capable of producing a similar affection (*Similia similibus*). Thus, Hahnemann adds : "So near had the great truth sometimes been approached, but only a hasty thought was here and there bestowed upon it. And hence the indispensable reformation of the ancient way of treating disease, the conversion of the traditional defective manner of treatment into a genuine, true, and certain art of healing remain unaccomplished to the present."

The old school claim their method of practice is rational because it is its aim to remove the cause of disease, and it follows the course of nature in her methods of getting rid of the offending presence. But, how can this "invisible disease power," this power intangible and beyond our knowledge, be affected by ponderable substances? We will see when we study the *Organon* proper that this "invisible (spirit-like) disease-power," can only be well met by a like invisible drug-power.

But to return. In a deranged stomach the old-school physician seeks to overcome the derangement by the use of medicines capable of combatting the present condition.

He believes the presence of the altered secretions in the stomach to be the cause of the sickness. And they are corrected, only to have the same conditions arise again after the action of the medicine is spent.

A patient may be affected with cancer, the offending growth is removed by the knife or external remedies used for the purpose, and the patient may be free for the time being, only to be overpowered by it later on, for the diseased condition itself, the products of the disease, and not the disease proper, was all that was removed.

Likewise, the chancre, when healed by cauterization, does not relieve the system of the poison within. The stopping of the gonorrhœal discharge, or later on removing the cauliflower excrescences only adds to the internal complication, and the external signs are removed. Very often, by this local interference, the disease is driven to some other part; or is forced to assume another form, and the change is attributed to the introduction of a new disease, while in truth it is the same disease, only altered in form because of the interference.

For example: If the too-free evacuation from the bowels be checked too suddenly by means of the old-school remedies generally used for such conditions, congestion of the brain may result.

The suppression of eruptions upon the skin by means of ointments, etc., often causes serious internal troubles to follow. All because nature was not allowed to dispose of the products of disease as she saw fit. The relief of rheumatic pains by using external applications often causes heart complications.

All these after-effects are far more serious than the original disease.

What could be farther removed from reason than the old method of blood-letting, salivation, excessive purgation, and overpowering glandular action, causing excessive perspiration and renal secretion?

When I was a boy, I knew a man about fifty or sixty years of age, who had not taken a step in many a day. He has told me his history, and it is this:

When a boy of about eight years of age he was taken sick. Before that time he was as bright and active as any lad, but after that sickness he lost the use of his lower limbs, and always walked upon crutches. He was not able to touch either foot to the ground, and all because of too much Calomel. From the hips upward he was a perfect specimen of manhood, but below the hips he was utterly helpless. The lower limbs were there, but they were like those of a boy a few years old, and without a particle of use to him. I have known of other persons but this man was a personal acquaintance.

Another method of treatment was the use of fontanels. An incision is made in the skin and some foreign substance is introduced into the opening for the purpose of creating an artificial ulcer, and by that means endeavoring to relieve the diseased body of the internal derangement through the artificial ulcer, but whatever relief came from this source soon vanished when the artificial ulcer was allowed to heal.

Likewise the internal disease is in no more danger of removal when Cantharides and other excitants are used upon the skin. The result is only a weakening of the vital powers. Still after centuries of progress many of their usages are counted the best that is known to medical science. Great is the mind of man!

Indeed we are living in a dangerous age—while we may have escaped the reign of the lancet, we are in the midst of bacteria of every description.

At this moment untold millions of these little pests surround us, only waiting the opportunity to overpower the system and lay us low with some one of the dread diseases. For my part, I cannot understand it. It seems to me that these specimens of minute animal life which the miscroscope reveals can only be the prodnct of some other poison.

I can only think they are a product of a poison already within the system, the same as the ordinary intestinal worms are the product of some internal ailment. There is that invisible something within the system which caused the first lumbricoid, and that invisible something being dispelled the product must necessarily disappear.

There is no statute law to prevent a physician from doing anything for his patient that may chance to come into his mind. The literature of to-day teems with accounts of unreasonable means used in the treatment of the sick. And if the patient chances to live the world is told of the wonderful result, and the attending physician is lauded for his heroism.

Of the uusuccessful efforts, we seldom ever hear.

But with the law of similars to guide us there is no need of resorting to questionable means. Be honest with yourselves, gentlemen. Begin at the foundation, and you will never regret in after years that you enlisted in the ranks for truth, and the most good to your fellow-men.

